# Comprehensive analysis of long noncoding RNA and mRNA in five colorectal cancer tissues and five normal tissues

**DOI:** 10.1042/BSR20191139

**Published:** 2020-02-14

**Authors:** Zhen-Xu Zhou, Xiao-Ming Chen, Yu-Qi Zhang, Liu Peng, Xiang-Yang Xue, Guo-Xin Li

**Affiliations:** 1 Department of General Surgery, Nanfang Hospital, Southern Medical University, Guangzhou 510515, Guangdong Province, China; 2 Department of Hernia and Abdominal Wall Surgery, The First Affiliated Hospital of Wenzhou Medical University, Wenzhou 325000, Zhejiang Province, China; 3 Institute of Glycobiological Engineering/School of Laboratory Medicine and Life Sciences, Wenzhou Medical University, Wenzhou 325035, Zhejiang Province, China; 4 Key Laboratory of Laboratory Medicine, Ministry of Education of China, School of Laboratory Medicine and Life Sciences, Wenzhou Medical University, Wenzhou 325035, Zhejiang Province, China; 5 Department of Microbiology and Immunology, Institute of Molecular Virology and Immunology, Institute of Tropical Medicine, Wenzhou Medical University, Wenzhou 325035, Zhejiang Province, China

**Keywords:** colorectal cancer, functional analysis, lncRNA profiling, mRNA profiling, target gene

## Abstract

The present study investigated the role of abnormally expressed mRNA and long noncoding RNA (lncRNA) in the development of colorectal cancer (CRC). We used lncRNA sequencing to analyze the transcriptome (mRNA and lncRNA) of five pairs of CRC tissues and adjacent normal tissues. The total expression of mRNAs and lncRNAs in each sample was determined using the R package and the gene expression was calculated using normalized FPKM. The structural features and expression of all detected lncRNAs were compared with those of mRNAs. Differentially expressed mRNAs were selected to perform Gene Ontology (GO) and Kyoto Encyclopedia of Genes and Genomes (KEGG) enrichment analyses. The functional analysis of differentially expressed lncRNAs was performed by analyzing the GO and KEGG enrichment of predicted *cis-*regulated target genes. A total of 18.2 × 10^8^ reads were obtained by sequencing, in which the clean reads reached ≥ 94.67%, with a total of 245.2 G bases. The number of mRNAs and lncRNAs differentially expressed in CRC tissues and normal tissues were 113 and 6, respectively. Further predictive analysis of target genes of lncRNAs revealed that six lncRNA genes had potential *cis*-regulatory effects on 13 differentially expressed mRNA genes and co-expressed with 53 mRNAs. Up-regulated CTD-2256P15.4 and RP11-229P13.23 were the most important lncRNAs in these CRC tissues and involved in cell proliferation and pathway in cancer. In conclusion, our study provides evidence regarding the mRNA and lncRNA transcription in CRC tissues, as well as new insights into the lncRNAs and mRNAs involved in the development of CRC.

## Introduction

A transcriptome is a collection of all transcripts in a cell, including messenger RNA (mRNA) and noncoding RNA (ncRNA), at a particular developmental stage or under certain physiological conditions. Transcriptome research is the basis for research on the function and structure of genes. Millions of reads can be obtained by high-throughput sequencing; sequence analysis can be performed by sequence alignment of these reads with reference genome based on existing gene annotation results. The differential expression of genes in different tissues, new transcript predictions, alternative splicing, single-nucleotide polymorphisms, and gene fusion can be analyzed by transcriptome sequencing [1–3]. Transcriptome sequencing technology is a highly flexible platform with a wide range of applications for high throughput, rapid, and high sensitivity analyses [4].

Long noncoding RNA (LncRNA) is a class of regulatory ncRNAs (length >200 nucleotides) found in multicellular animals in recent years [5]. LncRNA regulates chromosomal remodeling, genomic imprinting, histone modification, DNA methylation and RNA metabolism, as well as gene expression by *cis* and *trans* interactions with target molecules (e.g. DNA, RNA and proteins) [6]. The *cis* interactions affect the expression of neighboring genes based on gene location, while *trans* interactions refer to the ability of lncRNA to regulate the expression of encoding genes with the same expression pattern [7]. LncRNA forms an RNA regulatory network by regulating the expression of protein-coding genes, and participates in the physiological and pathological processes of the organism. Therefore, the change in the expression level of lncRNA is closely related to the occurrence and development of various diseases [8,9]. Annotation of eukaryotic transcriptomes by binding sequencing and biological information methods can reveal the functions of lncRNA in epigenetic modifications, transcriptional regulation and post-transcriptional regulation [10,11].

An increasing body of evidence shows that lncRNA is abnormally expressed in various types of cancer, regulating tumorigenesis, growth, apoptosis, invasion and metastasis, metabolism and other processes, which are closely related to the occurrence and development of various types of cancer [12,13]. The dysregulation of certain lncRNA target genes is also associated with the staging and prognosis of some tumors [14,15] characterized by tolerance to chemotherapy and resistance to targeted treatment [16,17].

The abnormal expression of lncRNA can be used as a potential biomarker for the diagnosis, prognosis and targeted therapy of cancer [18,19]. Transcriptome analysis can reveal abnormal expression or mutation of tens of thousands of lncRNAs related to tumorigenesis, metastasis, and tumor staging in various types of cancer [20].

Therefore, in the present study, lncRNA sequencing analysis was performed on five pairs of colorectal cancer (CRC) tissues and adjacent normal tissues. Functionalized enrichment analysis was performed on differentially expressed mRNAs and lncRNAs to determine whether their functional transcription was related to the development of CRC. Our data also contribute to a comprehensive understanding of the role of lncRNA in this process.

## Materials and methods

### Samples

Five CRC tissues and five adjacent normal tissues were collected from five patients with CRC who underwent tumorectomy at the Department of General Surgery, Nanfang Hospital, Southern Medical University (Guangzhou, Guangdong, China) between March 2015 and May 2016. These patients did not receive radiotherapy or chemotherapy prior to surgery and the tumor stage was confirmed using pathological methods according to the seventh edition of the Tumor-Node-Metastasis/American Joint Committee on Cancer classification. The patients included three males and two females, aged >70 years, with low or moderate tumor differentiation, and Tumor-Node-Metastasis stage II or IV (Table 1). Human cytomegalovirus testing and detection of related genes were performed as previously described [21]. Written informed consent was provided by all patients prior to the genetic research, and the research protocol was approved by the Ethics Review Committee of Nanfang Hospital, Southern Medical University.

**Table 1:** Clinicopathological features of colorectal cancer patients in the present study

No.	Gender^a^	Age	Location^b^	Differentiation^c^	T stage^d^	Pathology^e^	Tumor size	Number of lymph nodes	Metastasis^f^	TNM stage	HCMV(+)	UL47	UL56	UL77
1	1	75	0	2	3	1	6	0	0	II	1	1	1	1
2	1	73	1	3	3	2	5	0	0	II	1	1	1	1
3	2	86	3	2	3	1	4	4	1	IV	1	1	1	1
4	2	67	3	2	3	2	7	0	0	II	1	1	1	1
5	1	73	1	2	3	1	4	0	0	II	1	1	1	1

a: 1 is male, 2 is female; b: 0 is the rectum, 1 is the sigmoid colon, 2 is the right half colon, and 3 is the left half colon; c: 1 is high, 2 is medium, 3 is low; d: T1 is mucosa and submucosa; T2 is intrinsic muscular layer; T3 is subserosal/full-layer/outer serosa; e: 1 is ulcer, 2 is bulges; f: 1 is transferred, 0 is not transferred; HCMV(+): human cytomegalovirus, and 1 is positive; UL47, UL56, UL77 are HCMV genes, and 1 is positive.

### Sequencing and data processing

Total RNA was extracted and the ribosomal RNA was completely removed. Following the breakage of RNA into fragments, the sequencing adaptor was ligated and polymerase chain reaction amplification was performed to construct a sequencing library. Subsequently, sequencing was performed using an Illumina X10 with sequencing mode PE150. During pre-processing of the raw data, adaptor and low-quality sequences were removed through Cutadapt [22]. Data quality control was performed using FastQC [23]. Valid data were mapped reads to the reference genome using Bowtie 2 [24], TopHat 2 [25], and the mapped reads of each sample were assembled by StringTie [26]. A comprehensive transcriptome was reconstructed and the expression levels of all transcripts were estimated by StringTie and Ballgown [27]. The gene position information of the sequence was simultaneously counting according to the genome annotation file.

### Identification of mRNA and lncRNA

The comparison and visualization of the transcriptome were performed using the R package Ballgown (*P* < 0.01). The gene expression was calculated using normalized fragments per kilobase of exon model per million mapped reads (FPKM) values to avoid the effects of some false positives and extremely high-expression genes on low-abundance genes. The mRNA was analyzed at the gene and transcript levels. The known mRNA and transcripts <200 bp were removed, and the remaining transcripts were subjected to lncRNA prediction through coding potential assessment using Coding Potential Calculator (CPC) and Coding-Non-Coding Index (CNCI) software. These transcripts with coding potential (i.e. CPC score < −1 and CNCI score <0) were classified as novel mRNAs and filtered. The lncRNA sequence was finally obtained and genome mapping was performed to display the distribution of lncRNA candidates in the chromosome using the Circos software. Genome mapping was displayed according to different classification of lncRNAs in different samples.

### Comparison of mRNA and lncRNA

The structural features (i.e. length distribution, number of exons and length of open reading frame [ORF]) and expression of lncRNAs were compared and analyzed. The differences between lncRNAs and mRNAs were compared according to the ORF lengths of translation. The ORF analysis is mainly based on the six-frame translation principle of nucleic acids.

### Functional analysis of differentially expressed mRNAs and lncRNAs

The expression levels of mRNAs and lncRNAs were determined by calculating the FPKM [28] using StringTie. The differentially expressed mRNAs and lncRNAs who met the threshold of significant difference (*P* < 0.05) were selected using the R package-Ballgown [27]. Gene Ontology (GO) is an internationally standardized gene function classification system that provides a dynamically updated vocabulary to fully describe the properties of genes and gene products in organisms. The basic unit of GO is term (term, node), and each term corresponds to an attribute. The GO function significant enrichment analysis mapped all significant differentially expressed genes to the various terms of the GO database to calculate the number of genes per term. Subsequently, a hypergeometric test was applied to identify the GO terms enriched with differentially expressed genes versus the entire genome. *In vivo*, different genes coordinate with each other to perform their biological functions; pathway-based analysis assists in further understanding these biological functions of genes. Kyoto Encyclopedia of Genes and Genomes (KEGG) is the main public database for pathway analysis. The pathway significant enrichment analysis was also performed using a hypergeometric test.

### Prediction and functional analysis of differentially expressed lncRNA target genes

The *cis*-regulated target genes of lncRNAs are mainly predicted through their positional relationship. The lncRNA *cis*-regulation is defined as differentially expressed mRNA upstream and downstream (100 kbp range) of the differentially expressed lncRNA in the chromosome. GO and KEGG enrichment analyses were performed on the targeted mRNAs for lncRNAs using the BLAST2GO [29]. Statistical significance was denoted by a *P*-value <0.05. Pearson correlation analyses of differentially expressed lncRNAs and mRNAs were performed using the Co-LncRNA online software (http://bio-bigdata.hrbmu.edu.cn/Co-LncRNA/). LncRNA-mRNA pairs with correlation coefficient ≥0.8 and *P* < 0.05 were selected for the KEGG enrichment analysis. The lncRNA–mRNA network was performed using the Cytoscape software (version 3.6.1).

## Results

### Reads and mapping results of lncRNA sequencing

**Figure 1:**
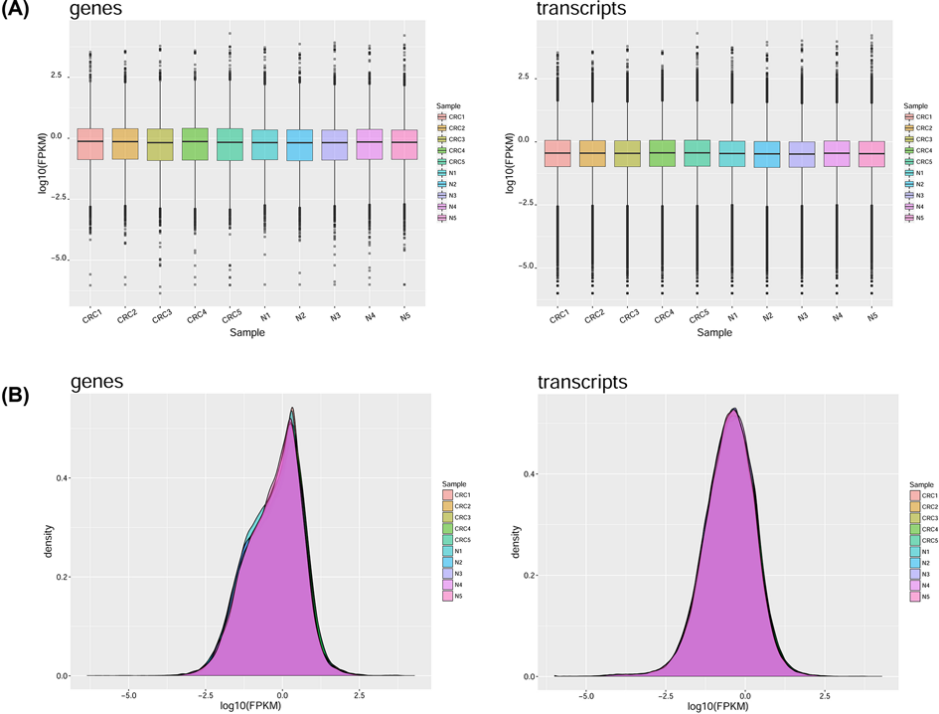
Gene expression analysis results of RNA sequencing (**A**) The distribution of gene expression was shown using a box plot: the abscissa is the sample name, the ordinate is log10 (FPKM), and the box plot of each region corresponds to five statistics (top to bottom are the maximum, upper quartile, middle value, lower quartile, and minimum). (**B**) Distribution of FPKM density. The expression density of each sample conforms to the normal distribution, and the expression trends of biological replicate samples are consistent. The abscissa is log10 (FPKM) and the ordinate is the density of the gene.

A total of 18.2 × 10^8^ reads were obtained by sequencing, in which the clean reads reached ≥94.67%, with a total of 245.2 G bases. The Q30 values in all samples reached >94.63% (Table 2). This sequencing amount and quality provided useful raw data for subsequent data assembly. The distribution of gene expression values, reflected in the FPKM box plot, indicated the repeatability of the design samples. The values of log10 (FPKM) were used to express the density of the expression values in different samples, which showed that the expression trends in different samples were consistent with normal distribution (Figure 1).

**Table 2:** Sequence statistics and quality control

Sample	Raw data		Valid data		Valid ratio (reads)	Q20 (%)	Q30 (%)	GC content (%)
	Read	Base	Read	Base				
CRC1	1.80E+08	27.00G	1.70E+08	25.71G	95.2	99.65	94.95	48
N1	1.50E+08	22.16G	1.40E+08	21.34G	96.29	99.66	94.96	50
CRC2	1.70E+08	25.41G	1.60E+08	24.31G	95.68	99.77	95.57	52.5
N2	1.90E+08	28.16G	1.80E+08	26.87G	95.44	99.74	95.51	49
CRC3	1.80E+08	26.34G	1.70E+08	25.21G	95.72	99.77	95.67	50
N3	1.80E+08	27.03G	1.70E+08	25.79G	95.42	99.73	95.12	49
CRC4	1.70E+08	25.96G	1.60E+08	24.72G	95.22	99.67	94.9	49
N4	1.70E+08	25.08G	1.60E+08	23.75G	94.67	99.64	94.63	50
CRC5	1.50E+08	23.11G	1.50E+08	22.13G	95.75	99.74	95.32	51
N5	1.80E+08	26.93G	1.70E+08	25.37G	94.22	99.79	95.6	52

**Table 3:** Differentially expressed lncRNAs and differentially expressed target mRNAs

	LncRNA						mRNA
Gene_id	Gene_name	Known/novel	Cis/trans	Cis location	Gene_id	Gene_name	Description
MSTRG.20579	CTD-2537I9.12	Known	Cis	100K	MSTRG.20574	ZNF865	Zinc finger protein 865
		Known	Cis	10K	MSTRG.20578	U2AF2	U2 small nuclear RNA auxiliary factor 2
		Known	Cis	1K	MSTRG.20578	U2AF2	U2 small nuclear RNA auxiliary factor 2
		Known	Cis	100K	MSTRG.20577	ZNF580	Zinc finger protein 580
MSTRG.11978	RP11-731F5.2	Known	Cis	100K	MSTRG.11978	IGHG4	Immunoglobulin heavy constant gamma 4 (G4m marker)
		Known	Cis	10K	MSTRG.11978	IGHG2	Immunoglobulin heavy constant gamma 2 (G2m marker)
		Known	Cis	100K	MSTRG.11987	IGHA1	Immunoglobulin heavy constant alpha 1
		Known	Cis	100K	MSTRG.11984	IGHG1	Immunoglobulin heavy constant gamma 1 (G1m marker)
MSTRG.30320	CTD-2256P15.4	Known	Cis	100K	MSTRG.30319	CCT5	Chaperonin containing TCP1, subunit 5 (epsilon)
		Known	Cis	10K	MSTRG.30319	CCT5	Chaperonin containing TCP1, subunit 5 (epsilon)
		Known	Cis	100K	MSTRG.30322	CMBL	Carboxymethylenebutenolidase homolog (Pseudomonas)
MSTRG.39692	RP11-229P13.23	Known	Cis	100K	MSTRG.39695	NPDC1	Neural proliferation, differentiation and control, 1
		Known	Cis	100K	MSTRG.39703	DPP7	Dipeptidyl-peptidase 7
		Known	Cis	100K	MSTRG.39696	ENTPD2	Ectonucleoside triphosphate diphosphohydrolase 2
		Known	Cis	10K	MSTRG.39691	SAPCD2	Suppressor APC domain containing 2

### LncRNA classification and comparison

**Figure 2:**
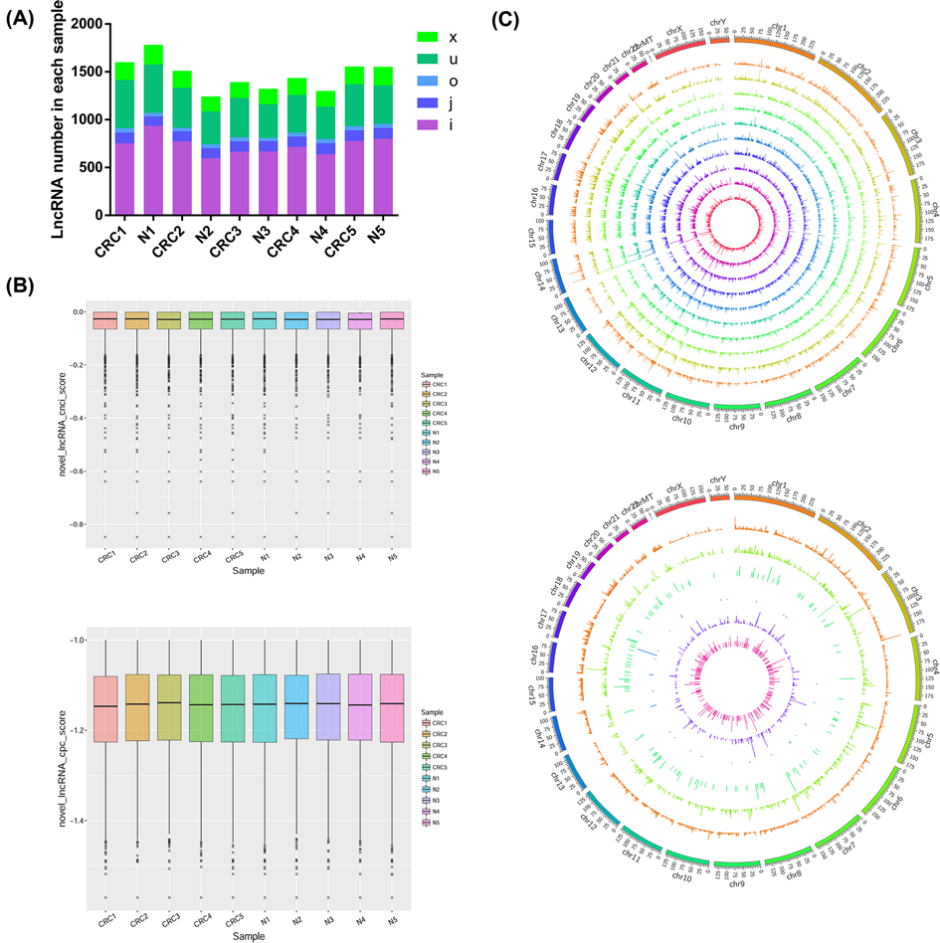
LncRNA screening analysis (**A**) A variety of lncRNA expression analyses. x, exonic overlap with reference on the opposite strand; u, unknown, intergenic transcript; o, generic exonic overlap with a reference transcript; j, potentially novel isoform (fragment); at least one splice junction is shared with a reference transcript; i, a transfrag located entirely within a reference intron. (**B**) The score statistic box plot of lncRNA CPC and CNCL in each sample. (**C**) Macroscopic statistics of the expression levels of lncRNAs in different chromosomes.

The lncRNAs predicted by CPC and CNCI were classified into five class codes: (1) potentially novel isoform (fragment), in which at least one splice junction is shared with a reference transcript; (2) a transfrag located entirely within a reference intron; (3) generic exonic overlap with a reference transcript; (4) unknown, intergenic transcript; and (5) exonic overlap with reference on the opposite strand. The distribution of various lncRNAs was similar in each sample (Figure 2A). The statistical results of FPKM expression in each sample showed that the expression levels of lncRNA in each sample were also similar, reflecting the good sample repeatability (Figure 2B). The Circos software was used to map the lncRNA obtained by screening and visually display the distribution of lncRNA candidates in the chromosome. The expression levels of lncRNA were similar in different samples, while the distribution of lncRNAs of different class codes in the whole genome varied (Figure 2C).

### Comparison of mRNAs and lncRNA

**Figure 3:**
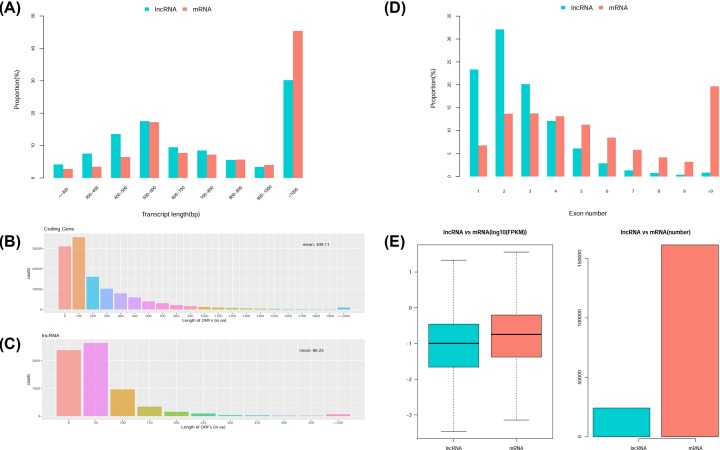
Structural comparison between lncRNAs and mRNAs (**A**) mRNA versus lncRNA length distribution. (**B**) mRNA ORF length distribution. (**C**) LncRNA ORF length distribution. (**D**) mRNA versus lncRNA exon number. (**E**) mRNA versus lncRNA expression level and number.

The length distribution of lncRNAs identified by sequencing was similar to that of mRNAs, mainly between 500–600 bp and >1000 bp (Figure 3A). The length of the ORF in the lncRNA sequence was mainly distributed between 50 and 250 amino acids, and the average ORF length was 86.25 amino acids. Notably, the length of the ORF in the mRNA sequence was markedly longer, with an average of 309.11 amino acids and mainly distributed between 100 and 1000 amino acids (Figure 3B,C). The number of exons in the lncRNA sequence was mainly between 1 and 6, while the distribution of mRNA exons demonstrated two peaks (i.e. 2–6 and >9) (Figure 3D). In terms of expression levels and numbers, those of mRNAs were higher than those of lncRNAs, especially in the latter (Figure 3E).

### Differentially expressed lncRNAs and mRNAs

**Figure 4:**
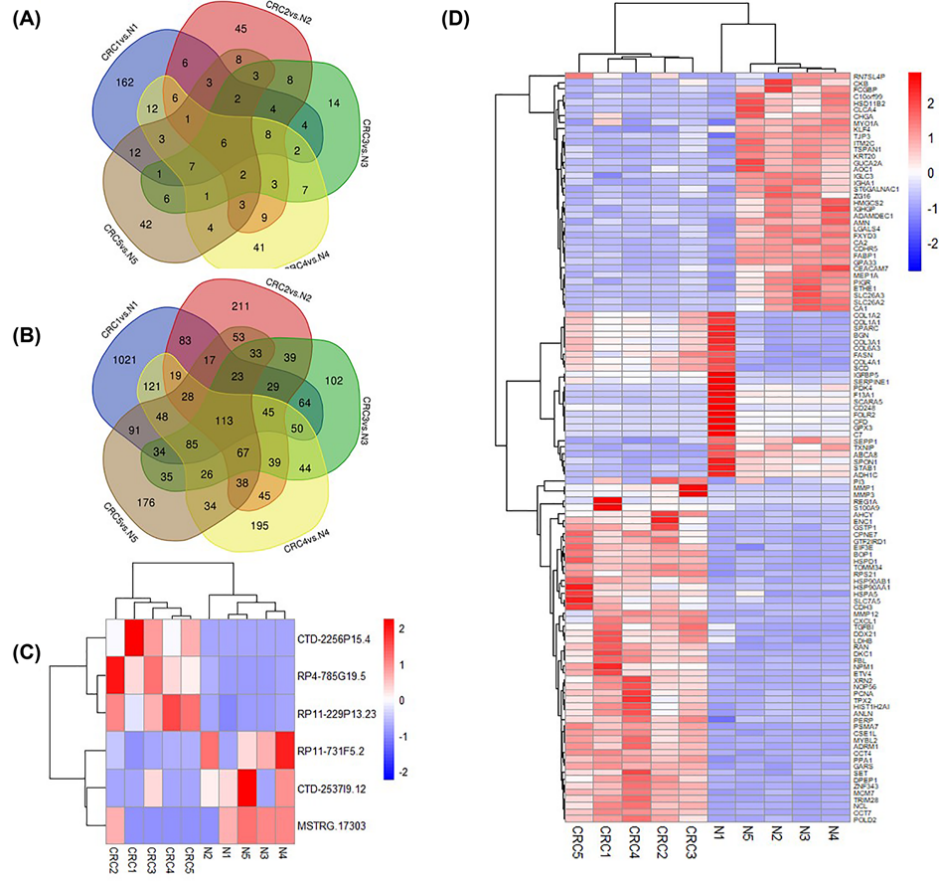
Differentially expressed lncRNAs and mRNAs in CRC tissues Venn plot of differentially expressed lncRNAs (**A**) and mRNAs (**B**) in five CRC samples. Cluster analysis of these lncRNAs (**C**) and mRNAs (**D**).

Difference analysis was performed on the assembled and quantified genes using the Ballgown package in R language. Finally, the number of differentially expressed lncRNAs detected in five pairs of samples was 239, 117, 78, 115 and 104, respectively. Of those, six lncRNAs (i.e. CTD-2256P15.4, RP4-785G19.5, RP11-229P13.23, RP11-731F5.2, CTD-2537I9.12 and MSTRG.17303) were abnormally expressed in all five CRC samples (Figure 4A) (Supplementary Table S1). The number of differentially expressed mRNAs detected in the five CRC samples was 1871, 882, 828, 997 and 901, respectively, and the number of intersections of the five data sets was 113 (Figure 4B) (Supplementary Table S2). Cluster analysis of differentially expressed lncRNAs and mRNAs was performed using the pheatmap package. The results showed that lncRNAs/mRNAs exhibited a large difference in overall expression patterns between CRC and non-CRC samples, while the expression patterns in the same group were similar (Figure 4C,D).

### Functional analysis of lncRNA target mRNA

**Figure 5:**
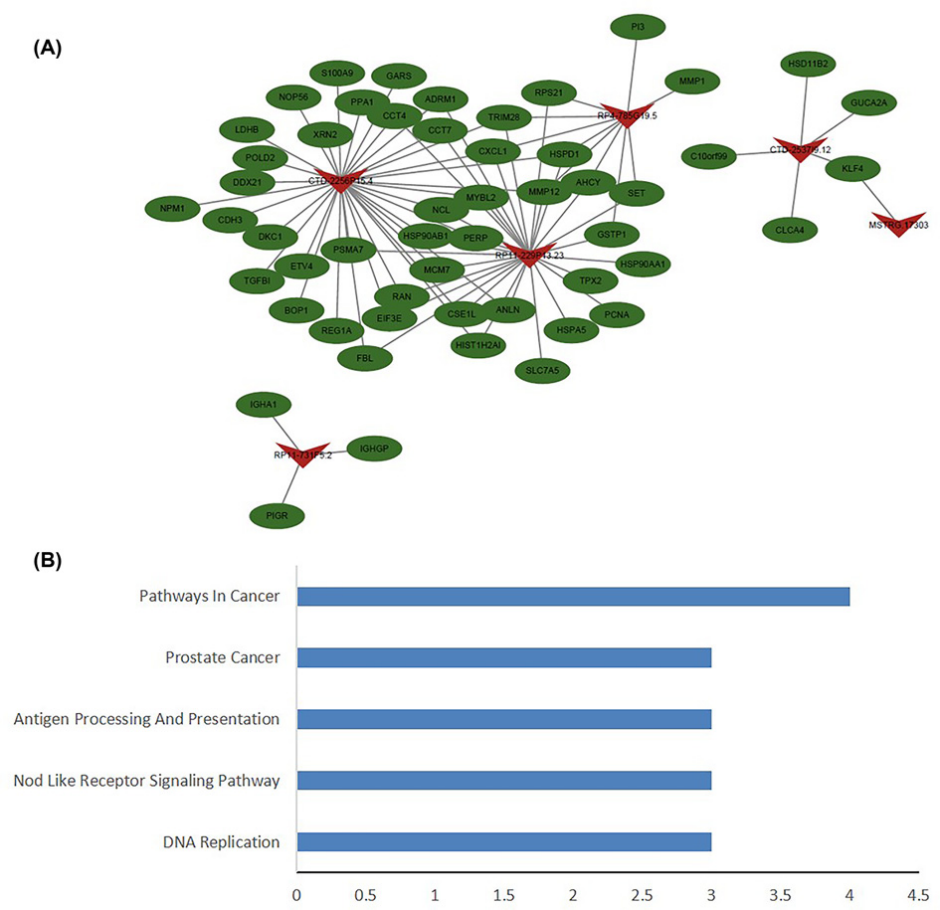
Functional analysis of differentially expressed lncRNAs in CRC tissues (**A**) The lncRNA–mRNA network was performed using the Pearson correlation analysis (correlation coefficient ≥0.8 and *P* < 0.05) and the Cytoscape software. Green denotes mRNA and red denotes lncRNA. (**B**) The KEGG pathway enriched by mRNAs co-expressed with lncRNAs.

A total of 15 differentially expressed mRNAs (*P* < 0.05) were screened by predicting the lncRNA *cis*-regulated target genes within 100 kbp upstream and downstream of the lncRNA (Table 3). The expression of these mRNAs was not consistent in the five CRC tissues. A total of 81 lncRNA–mRNA relationship pairs were obtained from the lncRNA–mRNA co-expression analysis. The lncRNAs with the highest correlation with mRNA expression were CTD-2256P.15.4 and RP11-229P13.23 (Figure 5A). The KEGG enrichment analysis showed that the main signaling pathways (*P* < 0.05) were DNA replication, nod-like receptor signaling pathway, antigen processing and presentation, prostate cancer and pathway in cancer (Figure 5B).

## Discussion

LncRNA is structurally similar to mRNA, without the function of encoding protein [30,31]. It can participate in the regulation of gene expression at the genetic level, transcription level, post-transcription level etc., and is closely related to the occurrence and development of various diseases [11]. The present study compared five CRC tissues and adjacent normal tissues through lncRNA sequencing. Bioinformatics comparison of the identified lncRNAs with mRNAs involved transcript length, exon number, ORF length, transcript number and expression levels. It was found that the transcript length, exon number, ORF length, number of transcripts and expression levels of lncRNAs were smaller/lower than those of mRNAs. This is consistent with the characteristics of lncRNAs reported in other studies [32,33].

The GO enrichment analysis of mRNAs with abnormal expression in CRC samples showed that differentially expressed mRNAs were mainly located in vesicles, exosomes, and the intracellular lumen, involving in protein localization and cytokine interaction. The peroxisome proliferator-activated receptors signaling pathway plays an important role in the regulation of cell differentiation, development, metabolism and tumorigenesis in higher organisms [34,35]. The present study found four abnormal genes in this signaling pathway (i.e. low expression of fatty acid binding protein 1 [FABP1] and 3-hydroxy-3-methylglutaryl-CoA synthase 2 [HMGCS2]; high expression of matrix metallopeptidase 1 [MMP1] and stearoyl-CoA desaturase [SCD]). FABP1 was found to block cytotoxicity by binding to harmful molecules, such as heme, fatty acids [36]. HCMV infection can cause increased mRNA level of MMP1 [37], and MMP1 can decompose the extracellular matrix and participate in tissue remodeling and metastasis [38]. SCD is an important metabolic control factor; inhibition of its expression enhances the therapeutic effects on many metabolic diseases [39]. This suggests that CRC cells may contribute to tumorigenesis by decreasing the expression of FABP1 and HMGCS2 and increasing that of MMP1 and SCD. The present study also found that collagen type I alpha 1 chain (COL1A1), COL1A2, COL3A1 and COL6A3, which are involved in the extracellular matrix-receptor interaction signaling pathway, are highly expressed in CRC tissues. COL1A1 and COL1A2 encode the alpha1 and alpha2 chains of type I collagen, respectively. It has been reported that the translocation of the COL1A1 gene is related to dermatofibrosarcoma protuberance, which is caused by the unregulated expression of growth factors [40]. This is consistent with the phenotype of patients with CRC. In addition, eight genes (i.e. calreticulin [CALR], protein disulfide isomerase family A member 3 [PDIA3], heat shock protein family A (Hsp70) member 5 [HSPA5], HSPA8, heat shock protein 90 alpha family class A member 1 [HSP90AA1] and HSP90AB1), involved in the antigen processing and presentation signaling pathway, were highly expressed in CRC. This indicated that protein processing was highly active in CRC tissue.

The results of the present study are inconsistent with those of other previous studies including data from The Cancer Genome Atlas. LncRNA GAS5, which is lowly expressed in CRC tumor tissues and plays a tumor suppressive effect [41–43], was highly expressed in CRC samples in the presdent study without reaching a significant difference in all pairs of samples. It has been reported that SATB2 antisense RNA 1 (SATB2-AS1) is down-regulated and associated with poor survival of patients with CRC [44]. However, this lncRNA was not abnormal in the present study. Up-regulated HOX transcript antisense RNA (HOTAIR), and oncogene colorectal neoplasia differentially expressed (CRNDE) [42], as well as down-regulated long intergenic non-protein coding RNA, p53 induced transcript (LINC-PINT), maternally expressed 3 (MEG3), PR-lncRNA-1 [43,45], PGM5-AS1, and B3GALT5-AS1 [46] were not detected in the present study. The specific reasons for this observation warrant further investigation.

The regulation of target genes by lncRNA can be divided into *cis* and *trans* according to the position relationship between lncRNAs and target genes in the genome [7]. Specifically, *cis* regulation mainly relies on *cis*-acting elements, which are sequences involved in the flanking sequence of genes and can affect gene expression. These elements (e.g. promoters, enhancers, regulatory sequences, inducing elements, etc.) are involved in the regulation of gene expression in the nucleus, and are usually transcribed into ncRNAs [6]. In the present study, a large number of lncRNA *cis*-acting target genes were predicted using software; however, not all target genes were abnormally expressed in CRC samples. This implies that the target genes are *trans*-regulated by lncRNAs or also regulated by other factors. However, differentially expressed mRNAs that co-expressed with differentially expressed lncRNAs in the present study were mostly involved in tumor-related signaling pathways and biological processes related to cell proliferation. These findings indicated that these lncRNAs may play an important role in the occurrence and development of CRC. This hypothesis should be further studied and verified.

In conclusion, we screened 113 differentially expressed mRNAs and six differentially expressed lncRNAs in five groups of CRC and adjacent normal tissues through lncRNA sequencing. Among those, 13 mRNAs were *cis*-regulated by lncRNAs and 53 mRNAs were co-expressed with these six lncRNAs. Finally, we performed GO function and KEGG enrichment analyses on these differentially expressed mRNAs. The results showed that these genes were involved in the occurrence and development of tumors by improving tumor cell activity, migration ability, and resistance to harmful factors.

## Supplementary Material

Supplementary Table S1 and S2

## Abbreviations

AS: alternative splicing
CNCI: Coding-Non-Coding Index
CPC: Coding Potential Calculator
CRC: colorectal cancer
FPKM: fragments per kilobase of exon model per million mapped reads
GO: Gene Ontology
KEGG: Kyoto Encyclopedia of Genes and Genomes
LncRNA: long noncoding RNA
PPAR: peroxisome proliferator-activated receptor
SNP: single-nucleotide polymorphism
