# Correction: Comprehensive analysis of long noncoding RNA and mRNA in five colorectal cancer tissues and five normal tissue

**DOI:** 10.1042/BSR-20191139_COR

**Published:** 2020-03-06

**Keywords:** colorectal cancer, mRNA profiling, lncRNA profiling, target gene, functional analysis

The authors of the original article ‘Comprehensive analysis of long noncoding RNA and mRNA in five colorectal cancer tissues and five normal tissues’ (*Bioscience Reports* (2020) 40(2); https://doi.org/10.1042/BSR20191139) would like to make several corrections to their publication.

1. In the sections ‘Functional analysis of differentially expressed mRNAs and lncRNAs’ and ‘Functional analysis of lncRNA target mRNA’, the authors would like to clarify that a threshold significance of |log2foldchange | ≥ 1 and *P* < 0.05 (noted also in their Supplementary Table) was used.
2. In their abstract, the authors had stated that ‘Further predictive analysis of target genes of lncRNAs revealed that 6 lncRNA genes had potential cis-regulatory effects on 13 differentially expressed mRNA genes and co-expressed with 53 mRNAs’. However, the results of the study only included the target genes of 4 of these lncRNAs. Furthermore, the number of selected mRNAs, cis-regulated by lncRNAs, was 15 (See Table 3 of the original article). Therefore this full sentence within the abstract should read ‘Further predictive analysis of target genes of lncRNAs revealed that 4 lncRNA genes had potential cis-regulatory effects on 15 differentially expressed mRNA genes and co-expressed with 53 mRNAs’.
3. Finally, tissue samples used in the study were taken from The First Affiliated Hospital of Wenzhou Medical University, not Nanfang Hospital, Southern Medical University.

The authors hope that these modifications provide better data uniformity, and apologise for any inconvenience that these errors have caused to the readers of the original paper.

